# Intervertebral disc cell- and hydrogel-supported and spontaneous intervertebral disc repair in nucleotomized sheep

**DOI:** 10.1007/s00586-012-2443-4

**Published:** 2012-07-29

**Authors:** Karin Benz, Claudia Stippich, Lisa Fischer, Klaus Möhl, Klaus Weber, Johann Lang, Frank Steffen, Barbara Beintner, Christoph Gaissmaier, Jürgen A. Mollenhauer

**Affiliations:** 1 NMI Natural and Medical Sciences Institute at the University of Tuebingen, Markwiesenstr. 55, 72770 Reutlingen, Germany; 2 Harlan Laboratories Ltd, Zelgliweg 1, 4452 Itingen, Switzerland; 3 Division of Radiology, Department of Clinical Veterinary Medicine, Vetsuisse Faculty Bern, Länggassstr. 128, 3001 Bern, Switzerland; 4 Department of Small Animal Surgery, Vetsuisse Faculty, Winterthurerstr. 260, 8057 Zurich, Switzerland; 5 TETEC AG, Aspenhaustr. 18, 72770 Reutlingen, Germany; 6 BG Trauma Center, Eberhard-Karls-University, Schnarrenbergstr. 95, 72076 Tuebingen, Germany; 7 Department of Biochemistry, Rush University Medical Center, Chicago, IL USA

**Keywords:** Intervertebral disc, Sheep injury model, Repair, Disc cells, Hydrogel

## Abstract

**Purpose:**

Regenerative repair is a promising new approach in treating damaged intervertebral discs. An experimental scheme was established for autologous and/or allogenic repair after massive disc injury.

**Methods:**

Disc healing was promoted in 11 animals by injecting in vitro expanded autologous/homologous disc cells 2 weeks after stab injury of lumbar discs L1-2. The following control discs were used in our sheep injury model: L2-3, vehicle only; L3-4, injury only; L4-5, undamaged; and lumbar discs from four non-experimental animals. Disc cells were suspended in a biologically supportive albumin/hyaluronan two-component hydrogel solution that polymerizes when inserted in order to anchor cells at the injection site. The parameters studied were MRI, DNA, glycosaminoglycan, collagen content, histology, immunohistology for collagens type I, II and aggrecan, and mRNA expression of GAPDH, β-actin, collagen type I, II, X, aggrecan, lubricin, and IL-1β.

**Results:**

All parameters demonstrated almost complete healing of the injured discs after 6 months, when compared with data from both the endogenous non-injured controls as well as from the healthy animals.

**Conclusion:**

Sheep experience spontaneous recovery from disc injury. The process of endogenous repair can be enhanced by means of hydrogel-supported cells.

## Introduction

Until now, prolapses of the intervertebral disc and disc degeneration are incurable disorders. Prolapse surgery relieves pain and pressure syndromes of the spinal nerves but could also aggravate or accelerate disc degeneration [1–3] resulting from inflammation and an impairment in the intrinsic healing capacity [4–7]. Autologous chondrocyte implantation (ACI) for large knee cartilage defects is now an established cell-based medical procedure [4], and, like bone marrow transplantation, most likely represents a key technology in regenerative medicine. It is conceivable to assume that, given the metabolic similarity of disc tissue to joint cartilage, the re-implantation of in vitro-activated autologous disc cells may exert a similarly successful approach to overcome those hurdles.

Unlike knee cartilage, however, intervertebral discs comprise at least two completely different tissues, the fibrous annulus with fibrochondrocytes and tendon-like fibroblasts, and the gelatinous nucleus with chondrocyte-like cells. Since it is practically impossible to separate each type of tissue from a harvested prolapse in the operating room, the question is whether a mixture of both cell types could induce healing [5].

In a knee ACI, cells are manually introduced into the open defect and anchored there, either by covering the defect with an impermeable membrane or by applying a carrier (matrix-supported ACI) [6, 7]. In autologous disc cell implantation (ADCI), such a supportive measure for targeted administration is not available yet and clinical applications rely on passive retention of injected cells at the site of injection; an assumption widely disputed, though.

Experimental clinical studies for ADCI have, so far, at least demonstrated the absence of untoward effects of the procedure and that may even hint toward a certain regenerative or at least protective effect in the patients [8, 9]. In risk assessments, however, it is essential to prove cell anchorage: a hydrogel formulation was thus developed allowing liquid injection directly followed by instant in situ gelation [10]. This hydrogel not only supports the survival and chondrogenic differentiation of various cell types, including articular chondrocytes, disc cells and bone marrow stromal cells (MSC) but also suppresses endothelial cells, bone cells and capillary formation [10, 11].

Although the anatomy of sheep and the biomechanics involved do not correspond fully with those of humans, sheep have been used in the context of intervertebral degenerative models. Experiments with these animals are inexpensive and, more importantly, are available in the biological age range matching that of adult humans. The type and extent of disc damage that should lead to relevant damage has also been described: the lesion must match the height of the disc and result in significant interruption of the annulus, thus creating a massive injury and causing the mechanical machinery of the disc to become labile [12, 13].

These factors were taken into account in inflicting the injury by means of a biopsy needle with a diameter close to that of the sheep disc [12, 13]. The biopsied tissue also served as a source for intervertebral disc cells. No discrimination was made between annulus and nucleus in order to mimic a putative harvest situation from a human prolapse. Cells isolated from these biopsies were expanded in vitro before being re-introduced into the damaged disc via injection. A polymerizing albumin-based hydrogel was used to anchor the injected cells in the disc. The animals were taken to be their own controls; the lumbar disc L1-2 was the pre-damaged disc that was given cell therapy, L2-3 served as the disc treated exclusively with the hydrogel, and L3-4 served as the damaged control. L4-5 was taken as the healthy control disc.

Besides the baseline biochemical composition data for collagen (via hydroxyproline), proteoglycan (via glycosaminoglycan) and DNA (cellularity), we analyzed gene expression at the mRNA level via real-time polymerase chain reaction (qRT-PCR). We gained data on major constituents in a healthy disc by analyzing the genes for collagen type I (alpha 2 chain), collagen type II, and aggrecan. Lubricin (PRG4) was added to that group because of a recent finding that underscores the importance of that molecule for proper function of the annulus fibrosus by providing for lubrication of interfibrillar sliding during annular shear loads [14]. Interleukin-1 served as an indicator of potentially accompanying inflammation. Collagen type X mRNA was taken as a sign of chondrocyte hypertrophy that typically develops as a result of disc degeneration [15].

The regular biochemical and histological patterns were evident in the discs after 6 months. In addition, MRI scans revealed that tissue properties returned to normal between week 6 and 3 months.

## Materials and methods

### Animals

Female sheep (Weisses Alpenschaf) were 2.5–3 years of age and weighed 51.3–72 kg. The animals were kept at Harlan Laboratories (Itingen, Suisse). The study was performed in an AAALAC-accredited laboratory (Association for Assessment and Accreditation of Laboratory Animal Care International), in accordance with the Swiss Animal Protection Law under license no. 374. In order to obtain baseline data, a group of four non-experimental sheep were killed and their corresponding lumbar segments were analyzed accordingly.

### Hydrogel preparation

The basic hydrogel component sheep albumin was activated as a maleimide derivative as described earlier [10].

To prepare 2 ml of hydrogel solution, 140 μl maleolyl-albumin (43 mM), 1,060 μl cell culture medium and 400 μl high molecular weight hyaluronic acid (20 mg/ml, Visiol, TRB Chemedica AG, Munchen, Germany) were mixed and incubated for 5 min at room temperature. The remaining 400 μl volume was reserved for the cell suspension. The crosslinker solution comprised 500 μl SH-PEG (10,000 g/mol, 15 mM SH-groups) in 0.1 mM HCl, to achieve a 1:1 ratio of maleolyl-groups to SH-groups when being mixed with the hydrogel/disc cell solution. The gel mixtures successfully underwent DIN/ISO 10993 biocompatibility testing (including gels prepared from the albumin of test species man, mouse, rabbit, rat, and guinea pig).

### Sheep tissue harvest/induction of damage

**Fig. 1:**
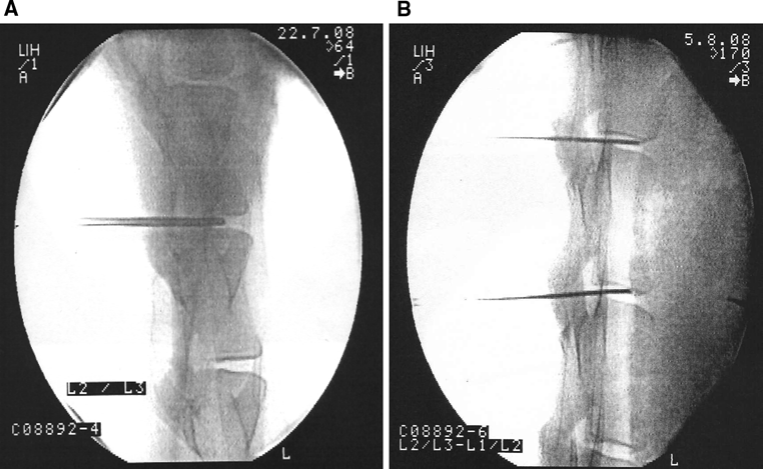
**a** C-arm X-ray image. 4-mm biopsy needle positioned into disc L2-3. The radiological image is a print out from the C-arm instrument, *perpendicular view*. **b** C-arm image: 18 gauge needles positioned into L1-2 and L2-3 prior to the injection

Anesthetized animals (butorphanol 0.1–0.2 mg/kg i.v./diazepam 0.1–0.2 mg/kg, followed by thiopental 15 mg/kg and then by 1 % propofol 5–15 ml, level maintained using isoflurane/oxygen) were positioned ventrally upright on the operating table, shaved in the lumbar spine area, with the area being disinfected with betadine^®^:water = 1:1. IVD tissue (consisting of combined annulus and nucleus tissue, as in human patients) was harvested under X-ray control using a 4-mm-diameter biopsy needle from discs L1-2, L2-3 and L3-4 (Fig. 1a). The needles were placed on to the discs, the core nail removed and the hollow needle inserted into the discs. The harvested tissue was transported in sterile containers to the tissue culture facility. In parallel, 50 ml of blood was drawn and converted to serum for the autologous tissue cultures.

### Cell culture

IVD cell isolation and culture was performed as described earlier [10], however autologous/homologous sheep serum was used instead of human AB serum. Isolated disc cells were plated in 75 cm^2^ cell culture flasks at an initial density of 0.1 million cells. Cells were cultured at 37 °C in humidified atmosphere containing 5 % CO_2_. The cells were harvested at 80–90 % confluence by trypsin–EDTA (BioWhittaker) treatment, washed by centrifugation, re-suspended in serum-free medium and counted including trypan blue for viability testing.

The sheep cells (for precise cell numbers, see Table 1) were suspended in the implantation culture medium (consisting of GMP grade phenol red-free medium, supplemented with 5 % serum, chondroitin sulfate, and BMP-2), then mixed with hydrogel solution and transferred into the 2-ml compartment of a dual chamber syringe. The second 0.5-ml compartment was filled with SH-PEG solution. The syringe was maintained at 4–10 °C until implantation, usually between 24 and 48 h (transportation and storage).

**Table 1:** Cell preparations for implantation in sheep: cell number and viability of the cells used for injection

Sheep no.	Cells/injection	Viability (%)
1. Homologous	500,000	99.9
2. Homologous	1,515,000	99.9
3. Autologous	445,000	99.9
4. Autologous	735,000	98.0
6. Homologous	420,000	97.8
7. Homologous	415,000	99.9
8. Autologous	420,000	99.9
9. Autologous	500,000	99.3
10. Autologous	500,000	99.9
11. Homologous	500,000	99.4
12. Autologous	2,060,000	99.9

### Surgical implantation into and recovery from sheep

The sheep received prophylactic antibiotic treatment: 30,000 IU penicillin/kg and 6 mg gentamycin/kg at 30 min prior to surgery and twice over 3 days postsurgically, plus tetanus prophylaxis 500 IU tetanus toxin/sheep. Animals were anesthetized and the area for operation was shaved and disinfected as before. The injection needles (18 gauge, 15 cm) for re-injecting the cells were pre-positioned under radiological control (C-arm instrument) (Fig. 1b).

After verification of the position through 90-degree rotation of the instrument, the radiation was shut down; and the dual chamber syringes were attached to the Luer lock of the needles. Injection was given simultaneously into L1-2 (gel plus cells) and L2-3 (gel only), slowly injecting approximately 0.5–1 ml gel solution, with a random decision being made on whether the animal would receive either an autologous or homologous cell preparation (see Table 1). Three animals received an “overdose” (No. 2, 4 homologous, No. 12 autologous) to monitor the potential effect of overdosing the cells in a disc. Six months after surgery, the animals were killed. The lumbar spine was collected, saw-cut longitudinally and the halves photographed and inspected for gross irregularities. The complete left part was fixed in buffered 4 % paraformaldehyde solution, and later processed for histology of paraffin-embedded disc segments. The right part was further dissected: the remaining halves of the discs were excised by knife, again cut to one-quarter IVD segments and shock-frozen in liquid nitrogen for further processing in biochemistry and molecular biology assays, respectively. Possible bias coming from this particular sampling procedure to cut the disc in pieces was accepted in order to generate as much data from one animal as possible.

### MRI radiology

Radiological examination was done at the Vetsuisse Faculty Bern, Division of Radiology, by means of sequential magnetic resonance imaging (MRI) inspections, starting with No. 1 prior to the tissue harvest; No. 2 after tissue harvest but prior to re-implantation of the cells; Nos. 3, 4, 5, 6, 7 immediately, 2 and 4 weeks, 3 and 6 months after implantation, respectively. Animals were imaged under anesthesia as described above. The images were taken using low field MRI (Hitachi Airis II-2, 0.3 Tesla, open, receiver coil with solenoid structure, position dorsal and parallel to lumbar spine, perpendicular to spinous processes of the vertebrae). Three sequences were applied: T2-weighted sagittal fast spin echo (FSE T2-w sag, TR 4,000 ms, TS 120 ms), T2-weighted transverse fast spin echo (FSE T2 tra, TR 4,698 ms, TE 120 ms), and T1-weighted gradient echo 3-D (1.2 mm slices, FE T1 MPR dors, TR 40 ms, TE 12 ms), with ventral saturation being present at all times. The images were assessed under blinded conditions (the radiologist was only given information about the spine segments to be assessed). Analysis criteria included the assessment of the width and signal intensity of the nucleus pulposus in T2-w sequences, definition of the endplates in T2-w and T1-w sequences, the boundary of the discs and the signal intensity (SI) of the epiaxial muscle. Grades ranged from 1 to 4 (one being best); and for calculation of the control level, the discs of one animal were compared and, in a second comparison, the animals were compared with each other.

### Histopathology

All tissue samples were processed and embedded into paraffin; 2–4 micrometer sections were made, and stained with hematoxylin-eosin (HE). In addition, selected sections were further processed and analyzed by means of immunohistology against collagens type I (anti-human collagen type I 63170, lot 1467 K, MP Biomedicals; LLC, Solon, Ohio, USA), II (antibody 2B5/ab3092, Abcam; Cambridge, UK), and aggrecan (SM1353, Lot 040308, Acris Antibodies GmbH; Herford, Germany). Secondary antibodies were supplied by the DAKO EnVision + System-HRP, with diaminobenzamidine used as a substrate. The stained sections were analyzed by normal light microcopy. Photographs were taken with a ColorView IIIu camera.

### Biochemistry

For the determination of DNA, glycosaminoglycans (GAG), and collagen, one-quarter pieces of IVD were digested with 1 mg/ml papain (Sigma-Aldrich) in 0.1 M Na-acetate, 0.01 M l-cysteine, 0.05 M Na_2_-EDTA, and 0.2 M NaCl (pH 6.0) at 60 °C overnight. The DNA content was determined using picogreen fluorescent dye (Molecular Probes/Invitrogen). Standard curves were generated at the time of each measurement using known concentrations of salmon sperm DNA (Eppendorf; Hamburg, Germany). DNA content was expressed as μg DNA/100 mg tissue wet weight. The GAG content was measured using the restrictive version of the dimethylmethyleneblue (DMB) assay, including guanidinium hydrochloride in the protocol [16] and with chondroitin-4-sulfate (Sigma-Aldrich) as a standard. Proteoglycan content was expressed as mg GAG/μg DNA or /100 mg tissue wet weight, as indicated. Total collagen content was measured by means of the hydroxyproline assay based upon alkaline sample hydrolysis, and reaction with chloramine-T and dimethylbenzamidine using gelatine as the standard [17]. The resulting values were expressed as mg collagen/μg DNA or /100 mg tissue wet weight, as indicated.

### Gene expression

To collect RNA from the explanted IVD, one-quarter pieces of IVD were ground in liquid nitrogen and the resulting fine powder instantly lysed in RLT buffer (Qiagen; Hilden, Germany). Total RNA was extracted using the RNeasy mini kit plus DNase I digestion according to the manufacturer’s instructions (Qiagen; Hilden, Germany). Complementary DNA (cDNA) synthesis and analysis of gene expression by semi-quantitative real-time PCR, using an Applied Biosystems 7500 Fast Real-Time PCR System, was done as described earlier [10]. Sequences of all primers used are summarized in Table 2. GAPDH and β-actin were used as reference genes. Ct value of the reference gene β-actin was subtracted from the Ct value of the gene of interest (dCt) and relative expression presented as 2^−dCt^.

**Table 2:** PCR primers used for gene expression analysis

Gene	Forward/reverse	Product length	Accession #
ACAN	GGGCAAGCTCCAGAAGCAA	124	FJ200438.1
	TTGGGAACCCAGATGGAAGTC		
COL1A2	TTTGTGGATACGCGGACTTTG	68	AY091602.1
	TGTAAGGGTTGGCATGTTGCT		
COL2A1	GGCAACAGCAGGTTCACATACA	78	FJ378650.1
	AATCACAGTCTCGCCCCACTT		
COL10A1 (Bos Taurus)	GGCCGTTTGTTAGTGCCAAT	143	NM_174634
	TTGGGTCGTAATGCTGTTGC		
IL1B	AGCCCGTCTTCCTGGGACGTT	98	NM_001009465.2
	CGTGGACCCCTGCGTATGGC		
PRG4 (Bos Taurus)	ACCTCCACCTCGGAGAATTACT	88	NM_001206633.1
	AGTTTTTCCTTCACAGTTGCATCTAGT		
ACTIN	ACGGGCAGGTCATCACCAT	95	NM_001009784.1
	GTGAATGCCGCAGGATTCC		
GAPDH	TGACCCCTTCATTGACCTTCAC	94	NM_001190390.1
	CCCGTTCTCTGCCTTGACTGT		

### Graphical presentation of data and data statistics

Data are presented applying the scientific software Sigmaplot v.11.0 (SPSS). Statistical analysis was performed within the same program, with the parameters indicated together with the datasets.

## Results

The sheep experiments were originally designed to reveal the modalities of disc healing in conjunction with cell therapy. As it turned out, and as described below, those goals had to be modified because we observed unexpected, spontaneous healing of the injured discs in the control groups as well. The dataset apparently describes the effect of cell therapy within a self-healing environment.

### Histology

**Fig. 2:**
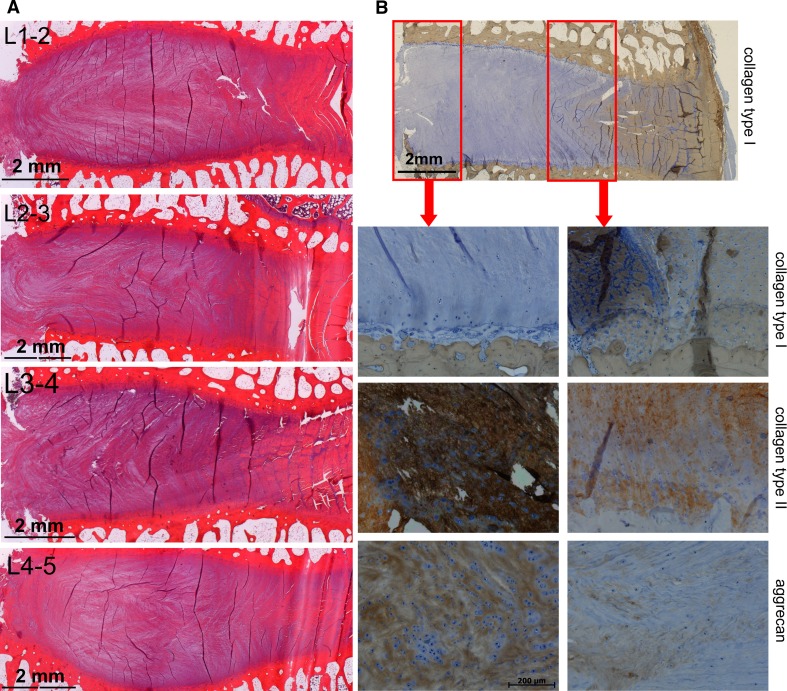
**a** Microscopic appearance of intervertebral discs, from *top* to *bottom* L1-2, L2-3, L3-4, and L4-5. Microscope magnification 1.25X. The *saw cut* is on the *left margin* of the images. The *dark lines* in the images are drying artifacts not related to disc features. **b** Immunohistology of key areas: stained with antibodies against collagen type I (low magnification survey and *second row*), collagen type II (*third row*), and aggrecan (*fourth row*). The *red boxes* indicate the areas of the core nucleus and the inner annulus zone, depicted as containing the enlarged areas below. Survey: 1.25X, details 10× objective magnification (see also scale bar). Positive immunostaining: *brown*; counterstaining: *blue*

On gross inspection, only two discs showed some alteration in appearance: in both cases, a slightly brown coloration that did not show a microscopic deviation from normal. The discs had a completely normal histological appearance. An intact eosinophilic annulus contained a hematoxylinic fibrous nucleus with elongated collagenous fiber structures. The sheep nucleus itself presented as a structure dominated by dissipated fibers apparently originating from the inner annulus, where they are more prominent in shape (Fig. 2a). Despite the structural relatedness of inner annulus fibers and the nuclear fibers, their biochemical composition is quite different. Immunohistology reveals the dominance of collagen type I in the outer annulus, a mixed fiber type from collagen type I and type II in the inner annulus, and a dominance of collagen type II in the nucleus. The large proteoglycan aggrecan is almost absent in the outer annulus and becomes gradually enriched towards the nucleus core where it dominates the space between the fibers and in between the disc chondrocytes (Fig. 2b). Histology confirmed MRI data which did not show any deviation from normal 3 months after disc injury. Two and 4 weeks post injury modest MRI signal reductions from the nucleus pulposus were found that disappeared subsequently. The signal changes did not relate to any particular treatment.

### Biochemical data

**Fig. 3:**
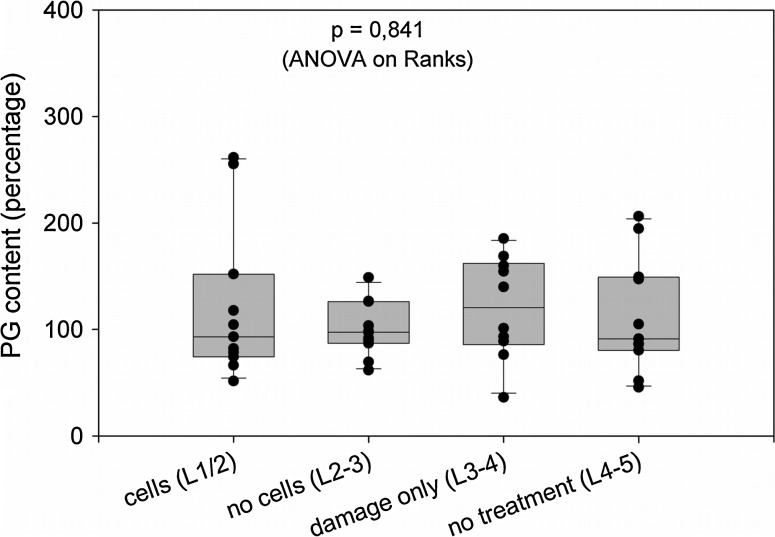
Glycosaminoglycan content of intervertebral discs. The data represent the relative amounts after calibrating data for wet weight in the individual discs and after installing the mean for the “no cells (L2-3)” group as 100 %. The *box plot* present the median (*line within the boxes*) and the 25/75 percentiles (*box sizes*), plus the standard deviations. The *dots* display the individual data for each of the 11 animals, with some dots overlapping. The data were statistically analyzed by one way ANOVA on ranks because distribution in most datasets was not normal (Kruskal–Wallis One Way Analysis of Variance)

There was no increased collagen content or glycosaminoglycan content in any treated disc. No increased DNA content was found indicating the absence of hypo- or hyper-cellularity in the discs. As an example, the glycosaminoglycan contents relative to the segments L2-3 are graphically presented (Fig. 3). The GAG content per wet weight was at approximately 4–5 %, and the collagen content at about 40 % in all discs examined (Table 3).

**Table 3:** Biochemical properties of the lumbar disc segments in the experimental group

Segment	DNA (μg/100 mg w/w)			GAG (mg/100 mg w/w)			Collagen (mg/100 mg w/w)			GAG/DNA (mg/μg)			Collagen/DNA (mg/μg)		
	Mean	Median	SD	Mean	Median	SD	Mean	Median	SD	Mean	Median	SD	Mean	Median	SD
L1-2	22.5	22.4	10.1	5.5	4.3	3.6	40.2	37.0	18.0	0.6	0.7	0.3	4.8	4.8	1.4
2-3	29.2	18.4	30.3	4.6	4.4	1.2	44.6	41.4	16.4	0.6	0.7	0.2	4.8	4.8	2.0
L3-4	25.2	20.9	19.2	5.7	6.7	2.2	45.0	45.4	14.6	0.8	0.8	0.4	5.6	4.4	1.6
L4-5	24.9	25.7	9.9	4.8	4.6	2.0	41.6	39.8	16.8	0.7	0.7	0.3	5.2	4.8	1.6

The data were statistically analyzed by one way ANOVA on ranks because the distribution of most datasets was not normal (ANOVA/Kruskal–Wallis One Way Analysis of Variance)

The data were statistically analyzed by one way ANOVA on ranks because most datasets were not normally distributed, as is evident in Fig. 3 and in Table 3, as the frequent differences between mean and medians indicate. This pattern seems to emerge due to the high individual variability of the animals rather than to a lack of measurement precision, the latter typically being below 5 % for the chosen DNA, GAG or collagen assay procedures.

The cellularity of the healthy animals was similar to that of the experimental group, with 10.7 ± 0.2 μg DNA/100 mg tissue wet weight comparing to 9.2 ± 3.4 for the experimental group. The mean overall proteoglycan content in the discs of healthy control animals was practically identical to that of the experimental group with 4.4 ± 0.35 mg GAG per 100 mg tissue wet weight. This was also true for the mean collagen content of 39.5 ± 1.7 mg per 100 mg tissue wet weight.

### Global mRNA expression data

**Fig. 4:**
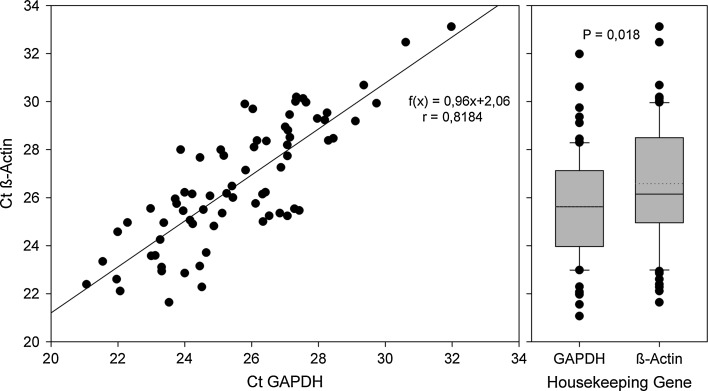
Analysis of the raw data for the housekeeping genes GAPDH and β-actin: expression levels per total RNA. The dataset includes the RNA preparations from all lumbar segments and represents the pooled data, irrespective of the underlying treatment of the segments. The *left portion* presents the correlation analysis; the *right graph* shows the gross difference of both genes expression levels. There is a slight but significantly higher level of β-actin expression per total RNA (*left*: Pearson product moment correlation, correlation coefficient 0.818, *p* = 1.65 × 10^−18^, 72 data pairs; *right*: two-tailed *T* test; *P* value = 0.0181)

Initially, the potential reference genes GAPDH and β-actin were compared for stability and suitability. We found a statistically significant, but biologically irrelevant difference in the expression levels of both genes; the raw data generated for both reference genes correlated well (Fig. 4). The data also document the reproducibility of the RNA preparation methods. In the subsequent analyses, β-actin was chosen as a reference gene.

**Fig. 5:**
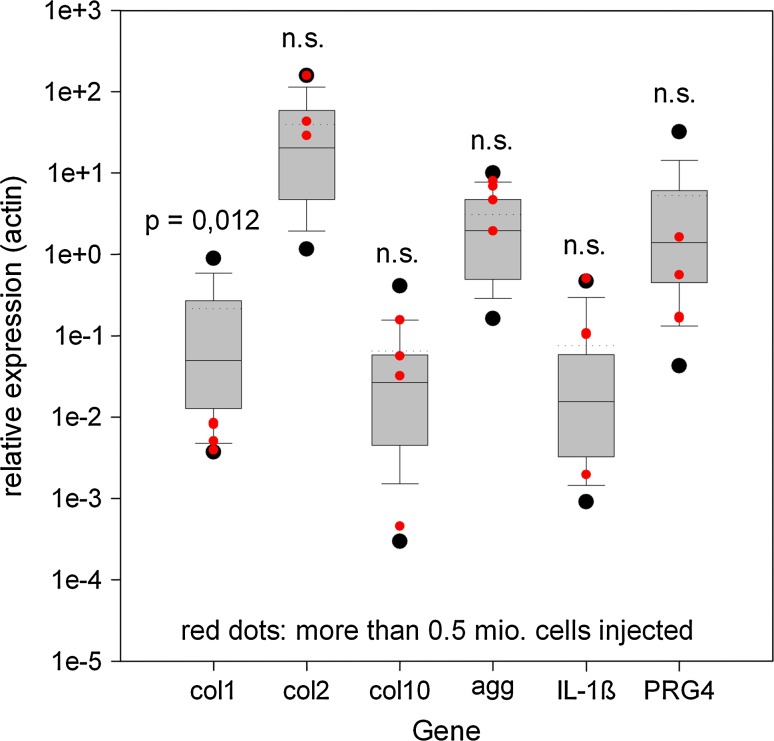
Global expression patterns for the genes analyzed in the study. As in Fig. 4, all data were pooled, irrespective of treatment or anatomical origin. The *red dot group* (increased cell numbers injected) is numerically included into the box plots. Because of the lack of statistical normality, the mean (*dotted lines*) and medians (*hairlines within the boxes*) differ in some groups (Mann–Whitney Rank Sum Test). Data are presented as 2^−dCt^ relative to β-actin expression

Figure 5 presents the global data for the quantified genes that were included in the study. The results demonstrate that the inter-individual variation was similar for all genes within the dimensional brackets of the relative expression levels. There was variation in the expression levels, across more than one order of magnitude, for every gene analyzed in the different animals. Typically, the relative expressions were high for collagen type II, aggrecan, and, surprisingly, lubricin (acronym for PRG4 [14]). As expected, collagen type X and IL-1β levels were low. We did not anticipate that the collagen type I level would be more than 100-fold lower (in comparison to collagen type II), despite there was no separation made between annulus and nucleus, indicating the prevalence of chondrocyte-like phenotypes within the entire disc. In the same context, no observable correlation was found between injected cell numbers for the cartilage genes and IL-1β; however the collagen type I expression was significantly (*p* = 0.012) lower in the segments that received more cells. The figures for collagen type II and aggrecan were slightly elevated—but not significantly—in those groups (Fig. 5).

The exact figures for the mean, medians and standard deviations for each treated lumbar segment are given in Table 4. The general lack of significance is mostly because the data obtained were not normally distributed. The expression in different lumbar segments showed some interesting trends; however they were not statistically significant because of the lack of distribution normality, as indicated by the occasionally large differences between mean and medians (Table 4). The control segment L4-5 exhibited the highest aggrecan expression levels. Collagen type II was high in L4-5 but highest in the cell-therapied L1-2. Collagen type X was highest in the damage-only group L3-4. The gel-only group had the highest levels of collagen type I mRNA and the lowest levels of IL-1β. Lubricin (synonym for PRG4) was prominent in L2-3, followed by L4-5.

**Table 4:** Experimental animals—relative gene expression data (normalized to β-actin)

Gene	L1-2			L2-3			L3-4			L4-5		
	Mean	Median	SD	Mean	Median	SD	Mean	Median	SD	Mean	Median	SD
COL1A2	0.17	0.02	0.26	0.57	0.32	0.93	0.27	0.05	0.43	0.27	0.02	0.08
COL2A1	65.20	28.70	64.90	48.30	31.20	35.70	42.10	18.10	49.20	53.8	39.90	44.80
COL10A1	0.13	0.06	0.16	0.15	0.03	0.30	0.39	0.05	0.62	0.21	0.06	0.24
ACAN	3.70	3.60	3.10	3.40	2.20	3.60	3.50	3.10	3.10	5.40	5.00	2.90
IL1β	0.12	0.02	0.19	0.05	0.03	0.08	0.09	0.02	0.11	0.14	0.05	0.21
PRG4	2.40	1.40	3.00	7.20	0.70	19.80	1.40	0.50	1.90	4.60	0.40	10.70

Data on mean values, medians and standard deviations (SD) are presented. No statistical significances were observed for any of the tested genes in the different treatment groups, respectively, disc segments (ANOVA/Kruskal–Wallis One Way Analysis of Variance)

The same type of analysis was done for the discs of healthy, non-experimental animals (Table 5). The individual variability across the disc segments and between the animals was the same as for the experimental group. In healthy animals, however, there were significantly lower expression levels for collagen type II (*p* = 0.002), aggrecan (*p* = 0.003), and for IL-1β (*p* = 0.002), but no differences were found for collagen type I, collagen type X or for lubricin (Kruskal–Wallis one way analysis of variance on ranks).

**Table 5:** Healthy control animals—relative gene expression data (normalized to β-actin)

Healthy animals (*n* = 4)	L1-2			L2-3			L3-4			L4-5		
Gene	Mean	Median	SD	Mean	Median	SD	Mean	Median	SD	Mean	Median	SD
COL1A2	0.07	0.04	0.08	0.17	0.19	0.12	0.14	0.08	0.16	0.10	0.10	0.02
COL2A1	7.80	2.76	11.50	6.30	6.50	6.10	12.50	7.30	14.80	6.20	2.20	5.40
COL10A1	0.01	0.01	0.01	0.01	0.01	0.02	0.04	0.01	0.06	0.01	0.01	0.01
ACAN	0.53	0.43	0.36	1.60	0.67	2.40	1.00	0.63	1.10	0.76	0.58	0.39
IL1β	0.002	0.002	0.001	0.005	0.004	0.003	0.033	0.003	0.061	0.005	0.002	0.007
PRG4	0.68	0.40	0.83	0.19	0.14	0.15	0.20	0.20	0.15	0.19	0.17	0.19

Mean values, medians and standard deviations SD are given. No statistical significance was observed for any of the tested genes in the different disc segments (ANOVA/Kruskal–Wallis One Way Analysis of Variance)

## Discussion

In its principle outcome, the experiment contains a surprising result: the more or less full spontaneous recovery of the injured sheep intervertebral discs. As this result was unexpected from the current situation described in literature, the applied strategy, aimed at demonstrating a cell-based regenerative measure, is compromised by the endogenous healing. Nonetheless, our data support the overall hypothesis that injection of disc cells embedded in a biological hydrogel may contribute to the regeneration of injured disc tissue. The histological datasets suggest that the repair processes occurred in a balanced manner: in general, neither under-repair (degenerated tissue structures, necrosis) nor signs of excessive matrix deposition (such as fibrosis) were observed. The data from gross biochemical analyses for collagen and glycosaminoglycan content underscore these qualitative findings.

Data on spontaneous recovery in the literature are difficult to compare. Three studies lasted for 3 months or less, 2 studies included 6 months and went on to 18 months. The gross and histological morphological changes described in those studies were modest and could not be observed in our study probably owing to the differences in the preparation of samples (halves instead of entire discs). However, the short-term studies present data that are also suggestive of regenerative events. Ahlgren et al. [18] report an increase of annular pressure after various annular incision injuries, taking place between 2 and 6 weeks after surgery. Schollum et al. [19] investigated annular lesions 3 months after injury and found discrete alterations of the annular fine structure. Fazzalari et al. [20] presented evidence of a reduction of disc stiffness 1 month after injury, that returned to normal at 3 and 6 months (statistical significance for month 1), but further increased at 12 months and again normalized at 18 months—all these trends were not statistically significant. Histology presented by Melrose et al. [21] indicates lasting effects at the incision site of the annulus, without obvious histopathology for the nucleus. A different model was applied by Zhou et al. [22], who damaged the disc by injecting the cell-poisoning compound Bromo-deoxy-uridine (BrdU). The study lasted up to 14 weeks. Enduring, prominent changes in the annulus and nucleus were reported, probably partly due to more than 50 % cell loss in the investigated areas.

In general, our quantitative data from healthy control animals and from the treated animals showed wide variability and distribution was not normal, making it necessary for statistics to be based on non-normal distributions, such as rank sum tests. There was variability not only among the animals but also between the four lumbar disc segments of a single animal. Histology was the only parameter that did not reflect these natural variations. It may be speculated that independent internal cycles of metabolic (anabolic and catabolic) activities form the basis for these variations. It is remarkable that the non-normality disappeared when the GAG- and collagen data were normalized to DNA rather than wet weight. This change in data quality could imply that the disc water content may fluctuate to some degree, whereas the ratio between cell number and extracellular matrix composition is more stringently controlled by the tissue.

In the global analysis of gene expression by means of PCR, the differences were as expected, with the mRNAs that define healthy intervertebral disc tissue being dominant. There were no signs of disc cell hypertrophy as the expression of collagen type X mRNA remained at the low level also observed for the pro-inflammatory cytokine IL-1. Somewhat surprising was the finding that collagen type I mRNA levels were rather low because the RNA has been extracted from the entire disc tissue and not only from the nucleus pulposus, the former containing significant quantities of collagen type I (see immunohistology data). The trends in the gene expression observed, when comparing the four disc segments, may indicate differences in the metabolism, stemming from the specific treatments, even though they occurred 6 months earlier. It was remarkable that the cell-therapied segment L1-2 had both the highest levels of collagen type II mRNA and the lowest levels of collagen type I mRNA.

Melrose et al. [21] had presented expression data for collagen types I, II, and aggrecan (among other genes) but displayed the data as a relative increase/decrease rather than as direct and housekeeping gene—normalized which makes them incomparable to outside datasets. However, they reported considerable and significant up-regulations of collagen type I and II mRNA in the damaged discs, as compared to controls, and a slight increase in aggrecan mRNA. We cannot confirm these findings, partly because of the high individual variations in our datasets and the different time course (3 vs. 6 months). Jünger et al. and Illien-Jünger et al. reported findings similar to ours in two subsequent papers: large (across more than two orders of magnitude) individual variations for collagen types I and II, and for aggrecan, albeit in an in vitro disc bioreactor model with up to 3 weeks in culture [23, 24].

The low expression level of collagen type X is not surprising, given the fact that there were no hypertrophic cartilage elements found in any histological sample.

In conclusion, the generated datasets suggest that biological repair of traumatic damage occurs in sheep intervertebral discs. The data also indicate that the injection of in vitro-expanded and hydrogel-supported autologous or allogenic disc cells into the damaged discs is possible, that this treatment does not compromise endogenous repair and may even be beneficial. The observed time course indicates that repair can take place in less than 6 months. We are currently conducting a second study which covers the time period from 4 weeks to 3 months.
